# Discovery of *Mycobacterium avium* subsp. *paratuberculosis* Lytic Phages with Extensive Host Range Across Rapid- and Slow-Growing Pathogenic Mycobacterial Species

**DOI:** 10.3390/antibiotics13111009

**Published:** 2024-10-27

**Authors:** Aleen Clare Golla, Jeanne Chaumontet, Rebecca Vande Voorde, Lia Danelishvili

**Affiliations:** 1 Department of Microbiology, College of Science, Oregon State University, Corvallis, OR 97331, USA; 2 National Veterinary School of Toulouse, 31300 Toulouse, France; 3 Department of Biomedical Sciences, Carlson College of Veterinary Medicine, Oregon State University, Corvallis, OR 97331, USA

**Keywords:** *Mycobacterium avium* subsp. *paratuberculosis*, Johne’s disease, lytic phages

## Abstract

Background/Objectives: Developing interventions for Johne’s disease, which focuses on controlling *Mycobacterium avium* subsp. *paratuberculosis* (MAP) in contaminated environments by treating infected cows and preventing transmission from diseased animals, is a critical priority. Bacteriophage (phage) therapy, an emerging biological intervention, offers a promising alternative for the treatment and management of MAP infections. Methods: In this study, we generated an MAP-specific lytic phage library aimed at characterizing the therapeutic potential of phages under environmental and biological conditions that mimic those encountered in infected cattle such as ruminal fluid, milk, colostrum, and the bovine intestinal epithelium, a key site of MAP colonization and, later, transmission. Results: Our library contains a diverse collection of phages that have demonstrated robust lytic activity against MAP. The host range of these phages was thoroughly assessed, revealing that several isolates produce clear plaques on a range of MAP strains, as well as other pathogenic non-tuberculous mycobacterial (NTM) species and *M. tuberculosis* strains. This broad host range expands the therapeutic potential of the phage collection, positioning it as a potential cross-species antimicrobial tool. In vitro tests under conditions replicating the rumen, milk, and colostrum environments show that selected phages maintain stability and lytic efficacy, even in the presence of complex biological fluids. Furthermore, a subset of these phages was capable of preventing MAP colonization and invasion in cultured bovine epithelial cells, suggesting their potential for direct prophylactic application in cattle. Conclusions. Our collection of MAP phages represents a valuable source that can be developed into probiotic-like preparations, offering a cost-effective solution for prophylaxis and control of Johne’s disease.

## 1. Introduction

*Mycobacterium avium* subsp. *paratuberculosis* (MAP) is a highly prevalent and contagious infection in dairy cows, commonly referred to as Johne’s disease. Due to MAP broad geographic spread in nearly every country, it poses a global threat to farmers. While the early infection stages of Johne’s disease make MAP diagnosis very difficult, the economic trade and distribution of silently infected animals further contribute to MAP dissemination in healthy herds. It is estimated that in the dairy industry, approximately 68% of herds are affected with Johne’s disease, and the economic loss due to increased cow replacement costs and reduction in milk production is estimated to be as high as $1.5 billion annually in the United States alone [1,2]. Additionally, while Crohn’s disease in humans is traditionally viewed as autoimmune in nature, mounting evidence suggests that it might have an infectious etiology, with MAP being implicated [3,4,5,6,7]. While the association between MAP and Crohn’s disease remains highly controversial, the mere possibility raises concerns regarding the potential risks to human health posed by the presence of MAP in milk and beef, as well as its contamination of natural water sources [8].

The current approach to deal Johne’s disease is very limited and has unsatisfactory results. Being unpreventable and incurable, there is a need for alternative interventions for the control, treatment, and prevention of Johne’s disease. Bacteriophages (phages) present one of the most promising new opportunities to fight infectious bacterial diseases [9,10]. Phages function like traditional antibiotics in a way that they exhibit powerful antimicrobial effect by infecting and lysing host bacteria. However, unlike traditional antibiotics, phages are highly selective and only kill organisms within a narrow taxonomic range. In recent years, phage therapy has gained attention as a promising treatment option for refractory, persistent, and disseminated infections in humans where antibiotics fail. Both in vivo studies and human clinical trials, including those involving pulmonary *P. aeruginosa* [11], and compassionate use in patients against pulmonary non-tuberculous mycobacterial infections [12] indicate that phage therapy is a safe, and often more effective, alternative to conventional antibiotic treatment [13,14,15,16].

Phages have been proposed for use in animal health, including veterinary applications [17], animal production environments [18], and more specifically, in the dairy production chain for prophylaxis, therapeutics, environmental decontamination, and dairy product bio-preservation [18]. Phage use in livestock has been mainly directed towards foodborne zoonotic infectious agents such as *Campylobacter*, *Salmonella*, *E. coli*, and *Listeria*. The landmark studies by Dr. Smith and his colleagues at Houghton Institute for Animal Disease Research (UK) that were carried out in young farm animals (calves, piglets, and lambs) led to a successful validation of the therapeutic use of bacteriophages against diarrheal diseases [19,20,21,22], demonstrating that severe experimental diarrhea in calves could be cured by a single dose of 10^5^ phage particles administered via an oral route, and, most importantly, can be prevented by a dose as low as 100 particles [22]. Multiple studies have explored the use of phages against *Campylobacter* in the poultry industry [23] and against *Salmonella* and pathogenic *E. coli* in the pig industry [24], providing strong evidence for significant reductions in intestinal colonized pathogen counts compared to mock-treated controls without causing any collateral effects on the gut microbiome [25].

Furthermore, phage preparations are used in food safety applications and in the animal production and processing industries to prevent pathogenic bacterial contaminations. Lytic phages effective against *Listeria monocytogenes* are used in ready-to-eat meats, processed fish, cheeses, frozen vegetables, and food contact surfaces [26]. While *Salmonella* phages have been applied in chicken and pork postharvest processing, *E. coli*-specific phages are used in cattle hide wash. Although phage-based products are not yet available for administration to animals, the FDA has granted provisional Generally Recognized as Safe (GRAS) status to food safety treatments against *E. coli* O157:H7, Shiga-toxigenic *E. coli*, Listeria, *Salmonella*, and *Shigella* using a single phage or phage cocktail [27]. Phage-based products such as Agriphage in the US and Biolyse in the UK are currently on the market. Also in the US, a company called Intralytics has launched bacteriophage products for poultry health: PLSV-1™, effective against Salmonella; and INT-401™, effective against *Clostridium perfringens* [28].

The majority of mycobacteriophages have been isolated in *Mycobacterium smegmatis* host [29,30]. However, limited studies report the discovery of phages active against MAP [31], as isolating such phages is extremely challenging due to the complexity of working with slow-growing bacteria and their tendency to clump. Consequently, the potential use of these phages in livestock and veterinary medicine has not yet been explored. In this study, we aimed to uncover MAP phages from diverse geographical regions and to create a lytic phage bank exhibiting activity against a range of animal and human MAP isolates. Our research objectives included characterizing these phages for the host range and virulence, assessing their stability under various environmental stress conditions and in the rumen environment. Additionally, we cross-examined the selected lytic MAP phages against a wide range of fast- and slow-growing mycobacterial strains and collected data on phage receptors. The potent phages of this study can be used toward the development of highly effective phage cocktails or probiotic-like additives for prophylaxis, early therapeutic interventions, and possibly use as bio-disinfectants in dairy farms. The discovery of MAP phages creates an opportunity to develop low-cost tools for Johne’s disease control and to support the health and welfare of agricultural animals.

## 2. Materials and Methods

### 2.1. Bacterial Strains and Culture Conditions

*M. avium* subsp. *paratuberculosis* K10 (MAP K10) was obtained from the American Type Culture Collection (ATCC). Additional MAP clinical isolates (Table 1) were sourced from ATCC or Dr. John Bannantine at the National Centers for Animal Health, IA. *M. smegmatis* mc^2^ 155 and *M. tuberculosis* (Mtb) auxotrophic H_37_R_v_ strains mc^2^ 6206 and mc^2^ 7901 were provided by Dr. William Jacobs, Albert Einstein College of Medicine, NY. *M. abscessus* clinical strains came from the Cystic Fibrosis Research Program at National Jewish Health, CO. Non-tuberculous mycobacterial (NTM) strains were supplied by Dr. Bermudez at Oregon State University, OR. *M. smegmatis* mc^2^ 155 was used for phage propagation, and NTM and Mtb strains were used for host range studies. All mycobacterial isolates were grown on Middlebrook 7H10 agar or in 7H9 broth with 5% OADC at 37 °C. For MAP strains, media were supplemented with 2 mg/L ferric mycobactin J.

### 2.2. Sample Collection and Preparation

Soil, soil–manure mix, and water samples were collected from North and South America and Asia under USDA permit P526-190513-011. Specimens were placed in 50 mL tubes filled to 25 mL. For solid samples, 20 mL of MP buffer (10 mM Tris/HCl pH 7.6, 100 mM MgSO_4_, 1 mM CaCl_2_) was added, followed by 24 h of gentle shaking. Samples were then centrifuged at 2500× *g* for 30 min, and the supernatant was transferred and centrifuged again. Sequential filtration through 1 µm, 0.45 µm, and 0.2 µm syringe filters followed. Water samples were similarly centrifuged and filtered. All cleared samples were spot-plated on 7H10 agar to check for contamination. Filtrates were stored at 4 °C.

### 2.3. Creation of MAP-Specific Phage Library

MAP K10 (ATCC BAA-968) was cultured at 37 °C on 7H10 Middlebrook agar supplemented with OADC and 2 mg/L ferric mycobactin J for 4–5 weeks. *M. smegmatis* mc^2^ 155 was grown to the mid-log phase over 3 days. Prior to phage isolation, bacterial suspensions were prepared in MP buffer, sonicated, and allowed to settle for 10 min. The single-cell preparation was then used for phage isolation experiments. Approximately, 100 µL of 3 × 10^8^ CFU (Colony Forming Units)/mL bacteria were incubated with 500 µL of cleared samples at room temperature (24 h for MAP, 30 min for *M. smegmatis*). Afterward, 4 mL of Top Agar was added and overlaid onto Middlebrook 7H10 agar plates (with or without Mycobactin J). Plates were incubated at 37 °C for 4–6 weeks for MAP and 3–5 days for *M. smegmatis* until plaques appeared. Plaques were excised, incubated in MP buffer, and re-propagated in *M. smegmatis* for higher titer recovery. Phages were purified using 100 kDa ultrafiltration membranes. Final phage titers were determined, and lysates (≥10^10^ PFU/mL) were stored at 4 °C.

Specimens not producing plaques on MAP K10 but yielding mycobacteriophages in *M. smegmatis* were re-tested on MAP using 10^10^ PFU titrations via spot testing and liquid culturing. In spot testing, 5 µL of phage lysates were applied to MAP lawns, while in liquid culturing, 50 µL of phage preparations were mixed with 10 µL MAP inoculum in 7H9 broth. Cultures were incubated for 7 days, with bacterial growth assessed via OD_600_ and CFU counts. The percentage reduction in bacterial growth was calculated relative to phage-free controls.

### 2.4. Phage Host Range in MAP Clinical Isolates

A phage library generated in MAP K10 was screened against additional clinical isolates of Johne’s and Crohn’s disease (Table 1). The host specificity was tested using a phage spot assay with standardized MAP inoculum (3 × 10^8^ CFU/mL). Two hundred microliters of inoculum were mixed with top agar and overlaid onto 7H10 agar plates supplemented with mycobactin J. Phage preparations (≥10^10^ PFU) were spotted (5 µL) onto the surface, and plates were incubated for 4–8 weeks or until plaque formation. Phages producing clear plaques were considered lytic against the MAP host. The assay was conducted alongside the MAP K10 reference strain. Additionally, a liquid culture test exposed 50 µL of phage (≥10^10^ PFU) to 10 µL of MAP inoculum (3 × 10^5^ CFU) in 200 µL 7H9 broth. After 7 days at 37 °C, bacterial growth was recorded at OD_600_, and CFU counts were determined. Bacterial reduction was compared to controls without phage, and the percentage of reduction was calculated.

### 2.5. The Efficiency of Plating (EOP)

The EOP was assessed for phages forming clear zones on MAP K10 across various clinical isolates. Phage preparations were standardized to 10^10^ PFU/mL, with 5 μL of each phage in 10–10^8^ titrations spotted onto agar overlays containing 200 μL of MAP at 3 × 10^8^ CFU/mL. Plates were incubated at 37 °C for 4 to 8 weeks until plaque formation was visible. EOP was calculated as the ratio of the phage titer on the clinical isolate to that on MAP K10. Phages were classified based on virulence: highly virulent (0.1 ≤ EOP ≤ 1.00), moderately virulent (0.001 < EOP < 0.1), and low virulence (EOP < 0.001).

### 2.6. Lytic Activity of MAP-Specific Phages Across Different Mycobacterial Species

The lytic activity of MAP-specific phages was evaluated against various mycobacterial species, including *M. abscessus*, *M. avium* subsp. *hominissuis*, *M. chelonae*, *M. fortuitum*, *M. kansasii*, *M. xenopi*, *M. gordonae*, *M. mucogenicum*, *M. bovis* BCG, and *M. tuberculosis* H_37_R_a_ and H_37_R_v_ auxotrophic strains. A standard plaque assay was conducted using phages at a titration of 10^10^ PFU. Two hundred microliters of each bacterial strain at 3 × 10^8^ CFU/mL was overlayed with 4 mL of top agar on 7H10 Middlebrook plates, and 5 μL of phages was stamped on the surface. Plates were incubated until clear plaques formed, taking approximately 3–4 days for fast growers and up to 3 weeks for slow growers.

### 2.7. Phage Temperature Stability

Active phages (10^10^ PFU) were serially diluted and placed in 96-well PCR plates. These plates were incubated at 43 °C or 55 °C for 5 h, with duplicate plates kept at room temperature as controls. An inoculum of *M. smegmatis* was prepared to a concentration of 3 × 10^8^ CFU/mL using McFarland Standard 1.0. Two hundred microliters of this inoculum was mixed with 4 mL of top agar and overlaid onto Middlebrook 7H10 plates. Five microliters of each phage dilution was stamped onto the agar surface. The plates were incubated until clear zones appeared, typically 3-4 days. Phage activity was evaluated based on the formation of clear zones relative to control plates, with lytic activity assessed by comparing phage reductions to control titration data.

### 2.8. Phage pH Stability

One hundred microliters of MP buffer at pH 5.0 was added to each well of a 96-well plate, followed by 10 μL of phage preparations (10^4^–10^10^). The phages were incubated in this acidic environment for 24 h. An inoculum of *M. smegmatis* was prepared at 3 × 10^8^ CFU/mL using McFarland Standard 1.0. Four milliliters of top agar was combined with 200 μL of the inoculum and overlaid onto Middlebrook 7H10 plates. Five microliters of the pH 5.0-exposed phages, as well as control phages incubated at pH 7.0, was spotted onto the agar surface. Plates were incubated until plaques formed. Lytic activity was determined by comparing plaque formation between the pH 5.0-exposed phages and the control phages, assessing stability and infectivity under acidic conditions.

### 2.9. Phage Rumen Stability

Ten microliters of phage preparations (10^4^–10^10^) was placed into wells of a 96-well plate, followed by 100 μL of freshly sterilized rumen fluid. The phages were incubated in rumen fluid for 24 h at 37 °C without agitation. Two hundred microliters of *M. smegmatis* inoculum at 3 × 10^8^ CFU/mL was mixed with 4 mL of top agar and overlaid on Middlebrook 7H10 plates. Five microliters of each phage preparation, including rumen-exposed and control phages, was spot-plated onto the agar. Plates were incubated until plaques appeared. Phage stability and efficacy were evaluated by comparing the plaque formation between rumen-exposed phages and control phages, determining the impact of rumen fluid on phage activity and infectivity.

### 2.10. Phage Stability in Milk and Colostrum

Freshly collected bovine milk and colostrum samples from Holstein Friesian cows were obtained. Phage preparations with titers ranging from 10^4^ to 10^1^0 PFU were dispensed in 10 μL aliquots into a 96-well plate. Each well received 100 μL of either milk or colostrum, and the plates were incubated for 24 h at 37 °C without agitation. After incubation, 200 μL of *M. smegmatis* inoculum (3 × 10^8^ CFU/mL) was mixed with 4 mL of top agar and overlaid onto Middlebrook 7H10 plates. Five microliters of each phage preparation, along with control phages incubated in MP buffer, was spot-plated. Plates were incubated for 3–4 days, after which phage stability and efficacy were assessed by comparing plaque formation between milk- and colostrum-exposed phages and control phages.

### 2.11. Mammalian Cell Culture and Colonization/Invasion Studies

Madin–Darby bovine kidney (MDBK) epithelial cells (CCL-22) obtained from ATCC were cultivated in Dulbecco’s Modified Eagle Medium (DMEM) supplemented with 10% heat-inactivated fetal bovine serum (FBS; Gemini Bio-Products, Sacramento, CA, USA) at 37 °C in 5% CO_2_. The bovine mammary epithelial cell line (MAC-T) was provided by Dr. Lewis Sheffield from the Department of Dairy Science at the University of Wisconsin. MAC-T cells were cultured in DMEM supplemented with 10% heat-inactivated fetal bovine serum, 5 μg/mL insulin, and 1 μg/mL hydrocortisone. MDBK and MAC-T cell monolayers (10^5^/well) in 96-well plates were exposed to selected phage at 10^10^ PFU for 30 min before adding MAP K10 inoculum (10^6^ CFU/well) at a ratio of 10 bacteria to 1 cell. The wells were lysed at 8 h and 24 h timepoints of MAP post-infection using 0.1% Triton X-100 (Sigma-Aldrich, St. Louis, MO, USA). The lysates were serially diluted in MP buffer and plated on 7H10 agar enriched with mycobactin J to quantify surviving MAP with CFUs in comparison to the MAP growth control of non-phage treatment over time.

### 2.12. Scanning Electron Microscopy

Approximately 10^8^ CFU/mL of MAP K10 strain was incubated with lytic phages (10^10^ PFU/mL) for 30 min or 4 h at room temperature, followed by microcentrifugation at 8000× *g* to pellet the bacteria. The supernatant was carefully removed, and the samples were fixed with 4% glutaraldehyde and 2% paraformaldehyde (Alfa Aesar, Ward Hill, MA, USA) for 30 min at room temperature. After fixation, the samples were microcentrifuged, the fixative was removed, and the bacterial pellets were resuspended in PBS. A 5 μL drop of each sample was placed onto a freshly glow-discharged, collodion, and carbon-coated grid. After 1 min, the grid was rinsed with 10 mM ammonium acetate and stained with 0.75% uranyl formate for 30 s. The samples were examined and imaged using a Helios 650 Ultra-Resolution Dual Beam FEG scanning transmission electron microscope at the Oregon State University Electron Microscopy Facility.

### 2.13. Phage DNA Preparation, Sequencing, and Genomic Analysis

Prior to DNA isolation, phage preparations were subjected to bacterial DNA and RNA removal. A 270 µL aliquot of phage samples (10^10^ PFU) was combined with 30 µL of DNase I 10× buffer, 1 µL of DNase I, and 1 µL of RNase. Samples were incubated at 37 °C for 1.5 h. To inactivate DNase I, 10 µL of 0.5 M EDTA was added, and the solution was incubated at 75 °C for 5 min. After eliminating host genetic material, the phage capsids were digested by adding 2.0 µL of Proteinase K (20 mg/mL) and incubating at 65 °C for 1.5 h. Phage DNAs were then purified using the GenElute Bacterial Genomic DNA Kit (Sigma-Aldrich, St. Louis, MO, USA) according to the manufacturer’s instructions. Phage DNAs were submitted to the Oregon State University Center for Quantitative Life Sciences Sequencing and Biocomputing facility and sequenced using the Illumina HiSeq 3000 system.

Gene prediction was performed using GeneMarkS and Glimmer, which provided the coordinates and FASTA amino acid sequences of predicted genes. These sequences were then compared against the NCBI BLASTp database to identify homologous genes. Full genome annotation was carried out using the DNA Master software 5.0.2 provided by the SEA-PHAGES program, which automated annotation based on BLASTn results from the NCBI database. A circular map was created using a Circular Genome Viewer (CGView) at http://wishart.biology.ualberta.ca/cgview (accessed on 25 September 2024) [32].

### 2.14. Phage Susceptibility to M. abscessus (MAB) Gene Knockout Mutants Involved in Surface-Exposed Glycolipid Transport and Synthesis

The MAB_0937c and MAB_0939 knockout mutants were screened for susceptibility via plaque formation assays using MAP phages. Inoculums of the MAB gene knockout mutants, along with the wild-type reference strain MAB 19977, were adjusted to 3 × 10^8^ CFU/mL. Then, 200 μL aliquots of the inoculums were mixed with 4 mL of top agar and overlaid onto Middlebrook 7H10 agar plates. Five μL of each phage preparation were spot-plated onto the soft agar surface and incubated for 4–5 days or until clear plaques became visible.

### 2.15. Statistical Analysis

To ensure reproducibility, most experiments were conducted for three independent trials, unless stated otherwise. Statistical comparisons between experimental and control groups were performed using Student’s *t*-test, analyzed with Prism 10 software (GraphPad). Significance levels were denoted as * *p* < 0.05 and ** *p* < 0.01.

## 3. Results

### 3.1. Isolation of MAP Specific Phages

**Figure 1:**
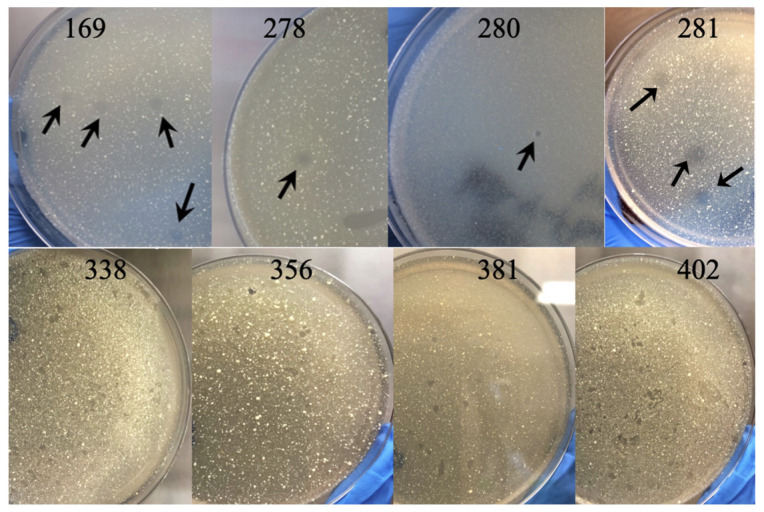
Identification of MAP phages with the double-layer agar plate method directly from environmental sample filtrates. The micrographs, captured at five weeks, display distinct and well-defined single plaques, some highlighted with arrows. These plaques are the result of lytic activity by eight different mycobacteriophages against MAP K10. Individual phage plaques were excised from the gel for subsequent propagation in *M. smegmatis*, achieving high titers of MAP-targeting phages (≥10^10^ PFU/mL).

To maximize phage discovery and diversity, specimens were sourced from dairy farms with a known history of MAP infections and from various geographical locations, climates, seasons, and environments, such as water and decaying soils exposed to manure. Through collaborations with veterinarians, veterinary technicians, DVM scientists, and students, we received over 1000 samples from the United States (Colorado, Florida, Hawaii, Minnesota, Nevada, North Carolina, New Mexico, Oregon, Texas, and Virginia), British Columbia, Chile, India, and the Philippines. Cleared samples were processed using the phage lysate spotting method on soft agar overlays of the MAP K10 strain, while the same filtrates were screened against *M. smegmatis* mc^2^ 155 to efficiently isolate mycobacteriophages. We identified eight lytic phages capable of forming clear plaques on MAP K10 (Figure 1) and 278 phages in *M. smegmatis* [33].

**Figure 2:**
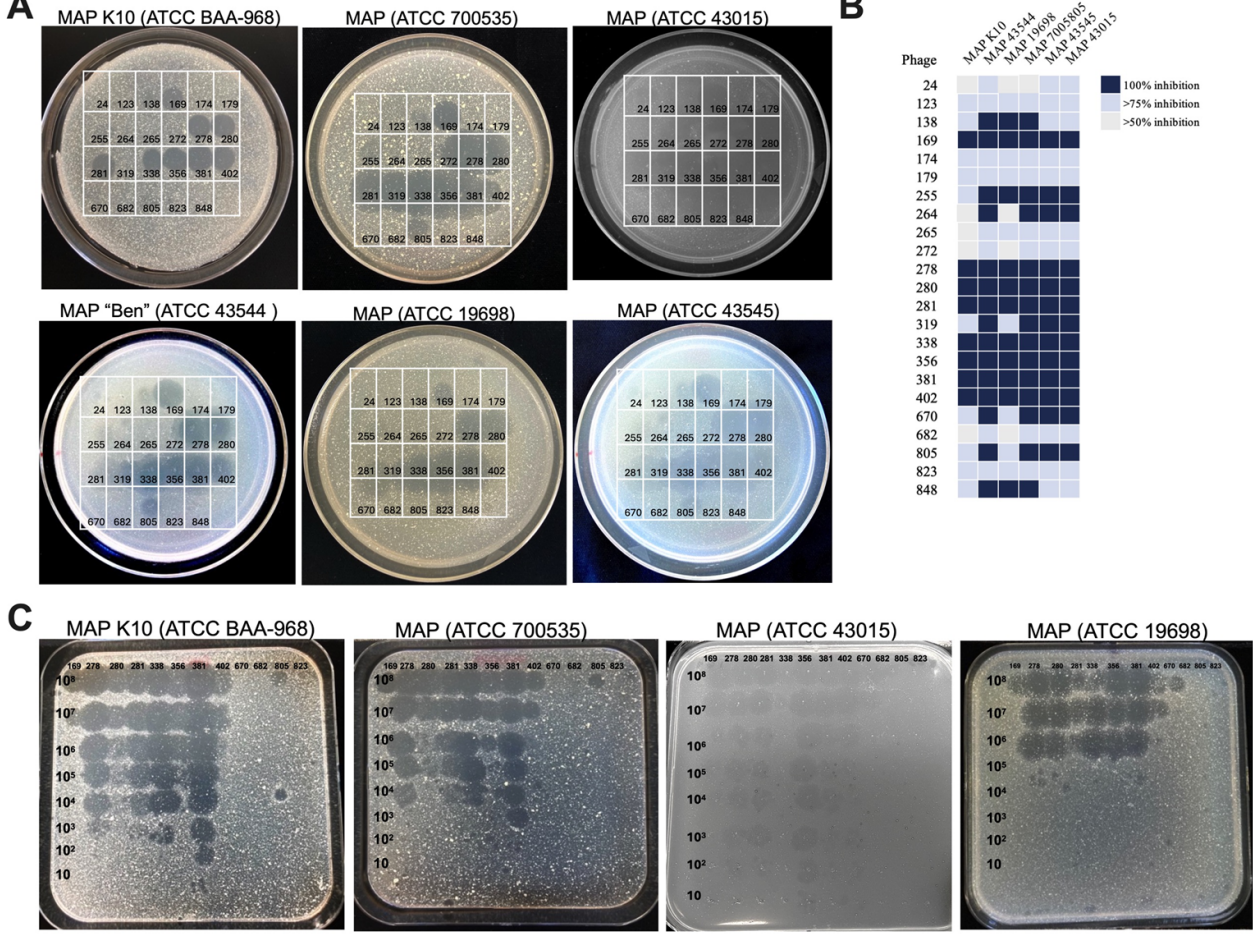
Phage host range and lytic efficiency in MAP strains. (**A**) Twenty-three phages were re-tested against MAP K10 and five additional clinical isolates using the double-layer agar plate method. While the bacteria formed a dense lawn, selected MAP-specific phages produced clear plaques and growth inhibition zones, confirming their lytic activity. Plates were incubated for up to eight weeks or until distinct plaques were observed. (**B**) The heat map summarizes the activity of twenty-three phages against six MAP isolates, assessed through liquid culturing methods. Phage efficacy was quantified by viable bacterial CFU counts, and the percentage of bacterial survival was calculated relative to control cultures without phage exposure. (**C**) Selected phages were further tested through titration to assess their virulence in titrations across different MAP isolates.

Given the possibility that the original samples contained very low concentrations of phages and that some active MAP phages may have been missed during direct screening, we re-tested the full library of 278 mycobacteriophages against MAP K10 at higher titers. Phage lytic activity was assessed using the spot testing method (Figure 2A) and liquid culturing through the CFU counts of viable MAP after 7 days of phage exposure (Figure 2B). Out of the 278 phages, 23 were found to significantly reduce viable MAP counts (≥50%). However, only 11 phages, including those originally isolated through direct screening on MAP, successfully produced clear plaques on the top agar overlays of the MAP K10 strain and/or fully cleared bacteria in liquid media (Figure 2A,B).

### 3.2. Characterizing the Host Range of MAP-Specific Phages

Twenty-three active MAP-specific phages were tested for their host range using a collection of animal and human MAP isolates, employing both spot testing and liquid culturing methods (Figure 2). Phages were classified as lytic if they completely lysed the host on soft agar (Figure 2A) and showed full clearance of bacteria in liquid culture (Figure 2B). The heat map illustrates the lytic activity and host specificity across six MAP strains. Most tested phages displayed broad activity against MAP isolates, with fifteen identified as lytic. For phage isolates that produced plaques on 7H10 agar overlays of MAP lawns, phage virulence was assessed through titration and compared to the sensitivity of MAP K10 (Figure 2C).

**Figure 3:**
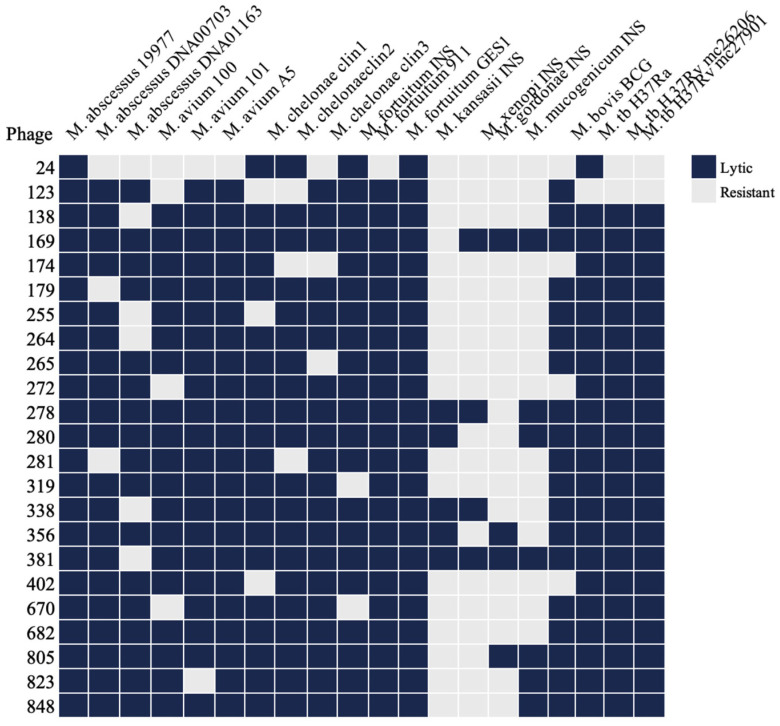
The lytic activity of MAP-specific phages across clinically relevant mycobacterial species. The heat map summarizes the lytic activity of twenty-three phages tested against 17 clinical isolates of various non-tuberculous mycobacterial species, as well as *M. bovis* BCG and *M. tuberculosis* (M. tb) strains. Lytic activity was determined using a plaque assay with the double-layer agar method at a phage concentration of 10^7^ PFU.

Additionally, selected phages were tested against various mycobacterial species, including *M. abscessus*, *M. avium* subsp. *hominissuis*, *M. intracellulare*, *M. chelonae*, *M. fortuitum*, *M. kansasii*, *M. xenopi*, *M. gordonae*, *M. mucogenicum*, *M. bovis* strain BCG, and *M. tuberculosis* H37Ra and auxotrophic H37Rv strains. Most non-tuberculous mycobacterial species and *M. tuberculosis* strains used in this study displayed susceptibility to a subset of phages, as illustrated in the Figure 3.

### 3.3. Evaluating Phage Stability Across Diverse Environmental Conditions of the Rumen

**Figure 4:**
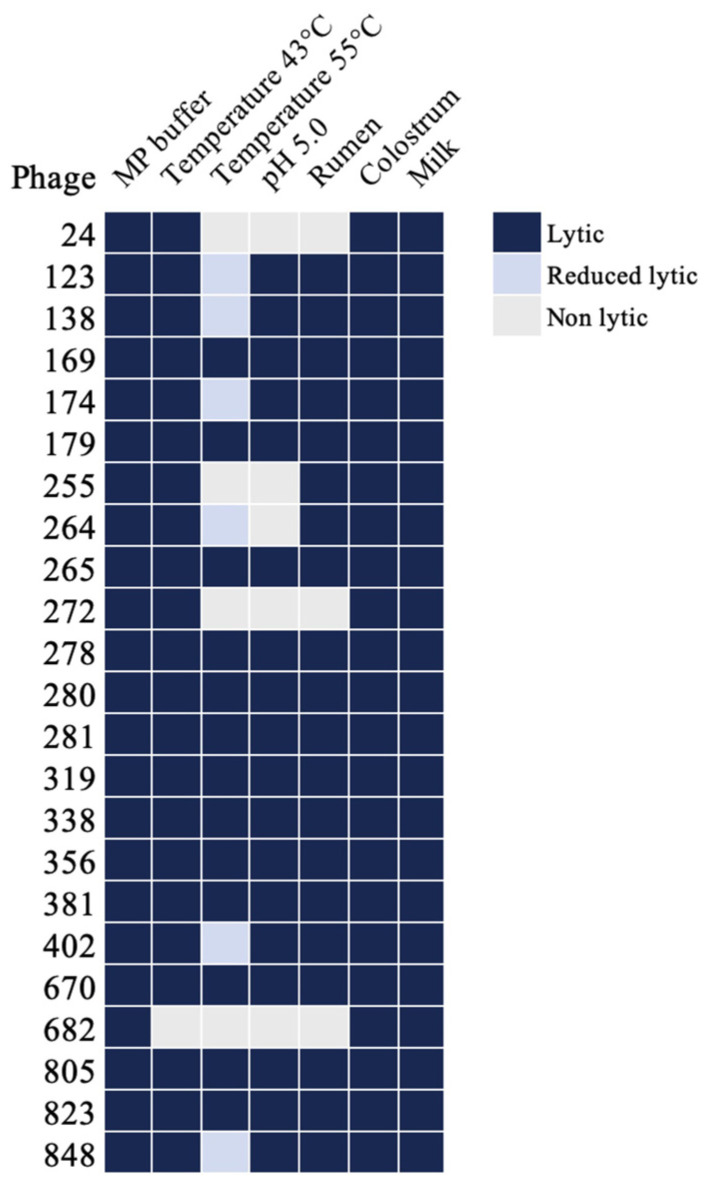
The impact of various environmental conditions on phage stability. The heat maps illustrate the lytic activity of phages under different conditions measured within a titration range of 10⁴ to 10⁹ PFU on *M. smegmatis* host. Phages were exposed to high temperatures or a low pH for 5 h or incubated in rumen fluid, milk, or colostrum for up to 24 h at 37 °C without agitation. After incubation, 5 μL of each phage preparation was spot-plated onto *M. smegmatis* overlays on Middlebrook 7H10 agar plates. The lytic activity of phages was evaluated by comparing plaque formation between phages exposed to various environmental conditions and control phages incubated in MP buffer. Lytic activity was defined as no significant difference in titers between the treated and control conditions. A reduction in lytic activity was defined as at least a 1 log decrease in titer, while non-lytic activity was characterized by a complete loss of plaque formation.

Evaluating phage stability in the cow rumen is essential due to its unique environment, characterized by temperatures exceeding 37 °C, low pH, and a complex mixture of anaerobic microbiota, ammonia nitrogen, fatty acids, and other metabolites. These factors can significantly impact phage activity, making it crucial to assess phage stability when administered orally through water or feed. We evaluated the stability of 23 phages under these conditions to determine their effectiveness in the rumen. Phage lytic activity was assessed on the fast-growing *M. smegmatis* host after 24 h of exposure to cow rumen fluid. Phage susceptibility was determined via spot testing, alongside a control group of *M. smegmatis* exposed to phages without prior conditioning. The results indicate that while a few phages lost lytic activity under certain conditions, most retained comparable activity to the control group (Figure 4).

### 3.4. Assessment of Phage Stability in Milk and Colostrum

Given the susceptibility of young animals to MAP infection through colostrum and milk from infected mothers, adding MAP phage cocktails could be a promising preventive strategy to protect calves from infection during feeding. Since milk and colostrum are rich in proteins, fats, carbohydrates, and other macronutrients and minerals, it is important to evaluate how these components might affect phage lytic activity. We incubated 23 selected phages in these environments and studied their activity in a titration range of 10^4^–10^10^ PFU in *M. smegmatis*. The results indicate that phage exposure to milk and colostrum did not affect its lytic activity when compared to the control group in MP buffer (Figure 4).

### 3.5. MAP-Specific Phages Effectively Prevent MAP Colonization and Invasion in Tissue Culture

**Figure 5:**
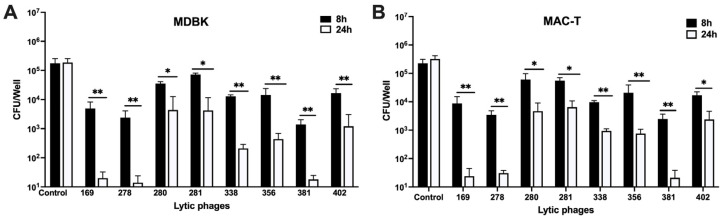
Phages effectively inhibit MAP colonization and invasion in epithelial cells. (**A**) MDBK or (**B**) MAC-T cell monolayers were pre-treated with phages at a concentration of 10^9^ PFU for 30 min before infection with MAP at an MOI of 10. Control wells, without phage pre-exposure, were included for comparison. At 8 h and 24 h post-infection, cells were lysed with 0.1% Triton X-100, serially diluted, and MAP counts were assessed by CFU enumeration following 4–5 weeks of incubation on Middlebrook 7H10 agar plates. The significance was determined using Student’s *t*-test (* *p* < 0.05, ** *p* < 0.01) within the treatment groups over time and between control and experimental groups. Data represent the mean ± SD of three independent biological replicates, each performed in quadruplicate.

This assay examined the ability of phages to inhibit MAP colonization and entry into bovine epithelial cells. MDBK cell monolayers were pre-exposed to selected phages at 10^10^ PFU for 30 min, followed by the addition of MAP K10 at an MOI of 10. Control wells without phage served as MAP growth controls. To account for the potential internalization of MAP by epithelial cells, MDBK cells were lysed at 8 h and 24 h, and bacterial CFUs were assessed. The results indicated varying degrees of inhibition, with three phages demonstrating significant early inhibition and complete clearance of MAP by 24 h when compared with control groups at corresponding times (Figure 5A). Five additional phages, while not completely eliminating MAP, still achieved a significant reduction in bacterial numbers at 8 h and 24 h timepoints. Additionally, the assay was performed on MAC-T bovine mammary epithelial cells (Figure 5B), yielding results consistent with those observed in MDBK cells.

### 3.6. Assessing Phage Attachment on MAP with Scanning Electron Microscopy

**Figure 6:**
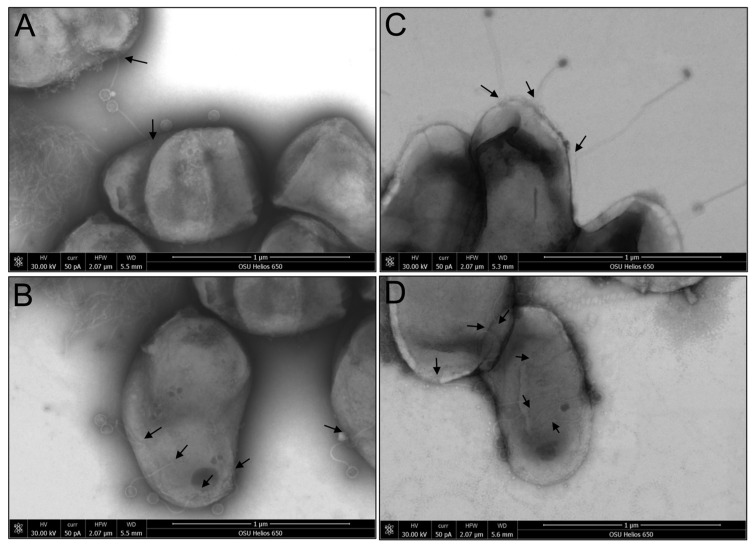
Scanning electron microscopy micrographs visualizing phage absorption on the cell wall of MAP. Panels (**A**–**D**) display four distinct phages (169, 278, 381, and 402, respectively) binding to the bacterial surface, as indicated by arrows. These phages can be identified by plaque formation on MAP, feature long, flexible tails, and are classified within the Siphoviridae family. They are characterized by their isometric heads and carry double-stranded DNA genomes.

MAP K10 was incubated with four different phages, three of which demonstrated the complete clearance of MAP in both in vitro assays and a bovine tissue culture model. SEM was used to visualize phage adsorption on the cell wall of MAP. The micrographs shown in Figure 6 reveal that selected phages began adsorbing to the bacterial surfaces as early as 30 min. In addition, SEM revealed that all four phages depicted in micrographs A through D belong to the Siphoviridae family, characterized by isometric heads and long, flexible, non-contractile tails, and are known to carry a double-stranded DNA genome.

### 3.7. Phage Genomic Analysis

**Figure 7:**
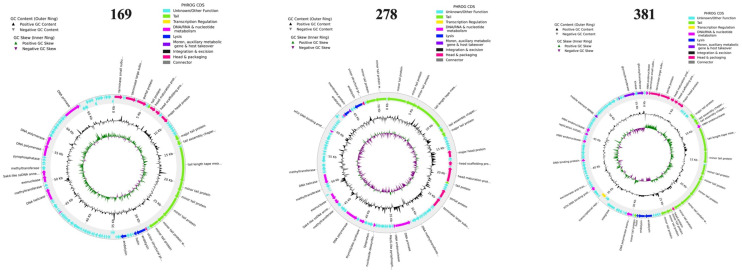
The genetic and physical maps of sequenced phages.

A comparative analysis of MAP-specific phages 169, 278, and 381 was performed using the complete nucleotide sequences through BLASTn in GenBank. The genetic and physical maps of sequenced phages are presented in Figure 7. Phage L169 has a genome size of 69.7 Kb, with 131 identified ORFs, 102 of which are annotated as hypothetical proteins. It exhibits a GC content of 48.6% and shares 98% sequence identity with phage MrMiyagi (GenBank Accession MT776806). Phage 278 has a genome size of 71.6Kb with a GC content of 56%, containing 118 ORFs, 84 of which are hypothetical proteins. It shares 99% sequence identity with phage Send513 (GenBank Accession JF704112). Phage 381 has a genome size of 56.6 Kb with a GC content of 61% and contains 115 ORFs, with 75 encoding hypothetical proteins. It shares 95% sequence identity with mycobacteriophage JalFarm20 (GenBank Accession MZ958744).

### 3.8. Establishing the Subset of Phages Utilizing Trehalose Polyphleates as Surface Receptors

**Figure 8:**
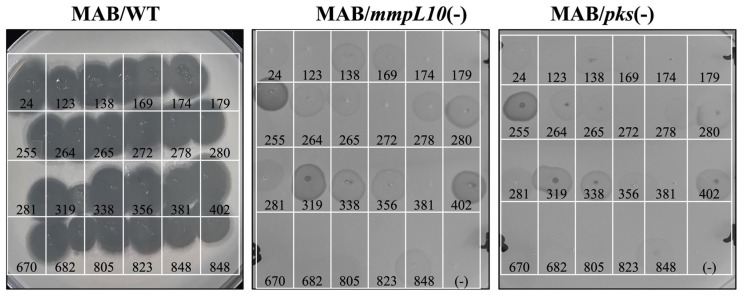
Phage resistance patterns in MmpL10 efflux pump and Pks mutants of MAB. The phage susceptibility assay, utilizing the phage plaque formation method, was conducted to evaluate the MAB 0937c/mmpL10 and MAB_0939/pks mutants in comparison to the wild-type MAB control.

Our previous research identified the surface-exposed glycolipids 2,3-diacyl trehalose (DAT) and polyacyltrehalose (PAT) as key targets for phage adsorption [33]. The MAB_0937c gene encodes the MmpL10 efflux pump, which exports these glycolipids to the bacterial surface, while the MAB_0939 gene encodes the polyketide synthase (Pks3/4) involved in DAT synthesis. Given that DAT and PAT are crucial components of the mycobacterial outer cell wall and essential in phage adsorption, this study utilized MAB_0937c and MAB_0939 gene knockout mutants to assess their susceptibility to MAP phages. Of the 23 phages tested, the absence of DAT/PAT resulted in partial resistance to 13 phages and full resistance to 10 phages (Figure 8), underscoring the importance of these glycolipids in phage adsorption mechanisms.

## 4. Discussion

The National Animal Health Monitoring System dairy study in 2007 revealed that 68% of U.S. dairy herds harbor *Mycobacterium avium* subspecies *paratuberculosis* infection in at least one cow, with nearly 100% prevalence in large herds [34]. It is also estimated that 8% of beef cattle herds are infected with MAP. However, because entire beef herds are not routinely tested unless an individual animal tests positive for MAP, Johne’s disease is likely more widespread in beef cattle herds than is currently recognized. The current approach to deal with the disease is very limited and of unsatisfactory results. Given the unpreventable and incurable nature of Johne’s disease, there is a critical need for effective strategies for its control, prevention, and treatment. In the absence of an effective vaccine, bacteriophages offer a promising alternative to combat MAP infections. Also, traditional antimicrobial agents for large animals are limited, and long-term drug administration for mycobacterial infections is impractical both physically and economically. In contrast, low-cost MAP-specific phages offer numerous advantages [10], including potential applications for treating both the early stages of Johne’s disease and later-stage infections in cows to prevent the spread of infection from diseased animals. Beyond treatment, phages can also be used as a ‘probiotic-like’ preparation and serve as a prophylactic intervention, administered periodically to healthy animals through water or feed additives to reduce the frequency of infections in dairy cows. This approach has the potential to make a significant impact on the industry. Due to the silent nature of MAP infections, which can take years to manifest as disease, administering phages to calves from birth via colostrum and milk could be the most effective strategy for preventing infections and reducing disease occurrence later in life. Additionally, one of the attractive characteristics of phages is their specificity, which could have a significant impact on controlling this environmental bacterium. Active phage preparations can be employed as biocontrol agents to reduce MAP infections by reducing bacterial loads in potentially contaminated dairy farm environments and lowering the overall risk of disease transmission.

The research on MAP phage identification and characterization is currently very limited [31] and, thus, their potential applications in livestock and veterinary medicine remain largely unexplored. To highlight, the majority of fully characterized bacteriophages against mycobacteria have been discovered in *Mycobacterium smegmatis* [29,30]. The isolation of MAP-specific phages is challenging, primarily due to the complexity of working with slow-growing bacteria and their tendency to clump. The primary goal of this research was to establish a robust phage bank targeting MAP and develop phage cocktails as cost-effective therapeutic and prophylactic interventions for Johne’s disease.

Phages that specifically infect MAP require a living host to replicate in natural environments, and MAP’s ability to survive long-term in these environments is well documented [35]. We hypothesized that environmental samples from dairy farms with a history of MAP infection would harbor lytic phages specifically active against this pathogen. To test this, we focused on manure-exposed sites, such as water sources and decaying soil, which are likely to yield phages with lytic potential against MAP. Samples were collected from diverse geographic regions and microclimates to maximize the likelihood of isolating effective phages. We also recognized that MAP-specific phages may exist at low titers due to the pathogen’s slow growth. To address this, we screened all purified environmental samples against *M. smegmatis*, a faster-growing surrogate host, to obtain phages at higher titers. These phages were then re-tested against MAP to enhance the chances of isolating specific phages. Rapid MAP-specific phage propagation and efficient production of therapeutic cocktails require the use of a fast-growing host. Leveraging *M. smegmatis* ensures the quick isolation and turnover of mycobacteriophages, crucial in developing effective phage-based treatments. *M. smegmatis* is a widely used model organism for studying various mycobacterial pathogens, including *M. tuberculosis* and the *M. avium* complex [36,37,38], making it ideal for phage isolation and propagation. Importantly, global collections of mycobacterial species-specific phages have been isolated using *M. smegmatis*, as all mycobacteriophages can infect this clade for reasons that remain unclear. This model organism replicates rapidly (every 1–3 h), enabling high-titer phage propagation in a cost-effective manner. Purified phage preparations from *M. smegmatis* are safe for use in humans and animals. In contrast, propagating phages in pathogenic strains like MAP would be slow, uneconomical, and unsafe due to the need for extensive purification to remove pathogenic contaminants, which could provoke adverse immune reactions. Additionally, *M. smegmatis* is a non-pathogenic commensal in humans and has been shown to act as a powerful cellular immune adjuvant [39,40,41,42,43].

The direct screening of sample filtrates on the MAP K10 strain identified eight MAP-specific phages that formed clear plaques on this slow-growing pathogen. Re-testing the *M. smegmatis* phage library on MAP K10 revealed 15 additional phages, which, while not all forming plaques, either cleared MAP K10 cultures or significantly reduced bacterial numbers. To develop an effective intervention for Johne’s disease, it is essential to identify MAP phages with broad-spectrum activity. In total, 23 active phages found in MAP K10 were tested on five additional MAP isolates. The results show that eight phages originally isolated on MAP K10 retained plaque-forming ability, with seven additional phages exhibiting lytic activity across other MAP strains, as demonstrated by clear plaque formation and/or bacterial clearance observed using a liquid culturing method. Notably, phages capable of forming plaques suppressed bacterial growth for over two months throughout the experiment. Of the 23 phages discovered in the MAP K10 strain, most exhibited activity across various MAP clinical isolates, significantly reducing bacterial loads in vitro. Additionally, we tested these phages against a wide range of NTM species and *M. tuberculosis* strains, finding that some MAP phages demonstrated an extensive host range, with lytic activity against the majority of tested mycobacterial species and strains. The broad host range of this phage collection enhances its therapeutic potential, making it a promising antimicrobial tool for use across multiple species.

Orally administered phages face challenges in the bovine digestive tract due to high temperatures and acidity, which can affect their stability. The rumen, with its complex chemical composition and rich anaerobic microbiota, can also impact phage activity. For prophylactic applications in calves, phages must remain stable and virulent in milk and colostrum. In vitro results demonstrate that the majority of phages tested are stable and maintain lytic efficacy under various stress conditions, including those present in the bovine intestine, rumen, milk, and colostrum. Additionally, tissue culture studies identified robust phages that prevent MAP colonization and invasion in bovine epithelial cells by clearing or significantly reducing viable bacteria.

The diversity and versatility of phages are key advantages for therapeutic applications; however, an important aspect of designing effective phage preparations is mitigating the development of phage resistance in bacteria [9,44]. To achieve this, it is essential to create a cocktail of lytic phages that can bind to different host receptors and employ various mechanisms for absorption into bacterial cells. In this study, we sequenced three phages, all identified as Siphoviridae morphotypes through SEM imaging, but belonging to different genetic clusters of AC, F, and R based on comparative analysis. Moreover, building on our previous discovery of DAT/PAT trehalose polyphleates being the critical binding targets for a subset of phages in *M. abscessus* [33] and supported by findings from other studies [44], we explored their role in MAP phage adsorption, as well. Using MAB knockout mutants lacking the MmpL10 efflux pump or polyketide synthase, we assessed susceptibility to MAP phages, distinguishing those reliant on trehalose polyphleates from those with independent lytic activity.

In this study, we successfully isolated and characterized 23 lytic phages targeting MAP. This work lays a strong foundation for expanding the MAP phage research, particularly in designing phage cocktails that could be used both to prevent and treat Johne’s disease in infected animals, aiming to reduce the infection transmission to healthy animals. The MAP-specific phage bank represents a valuable resource for cost-effective probiotic-like product development, offering targeted and sustainable solutions for one of the most persistent and costly diseases in dairy farming.

## Figures and Tables

**Table 1:** The list of *M. avium* subsp. *paratuberculosis* clinical isolates used in this study.

Strain Designation	Source	Isolation Source
MAP K10	ATCC BAA-968	Animal feces
MAP “Ben” [CIP 103966]	ATCC 43544	Intestinal tissue of Crohn’s disease patient
MAP 97R0816	ATCC 700535	Mesenteric lymph node extracted at necropsy from 6-year-old Guernsey cow with a 3-year history of Johne’s disease
MAP “Linda” [CIP 103965]	ATCC 43015	Ileum of girl with Crohn’s disease
MAP19698	Dr. John Bannantine	Animal feces
MAP “Dominic” [CIP 103967]	ATCC 43545	Intestinal tissue of Crohn’s disease patient

## Data Availability

Data are contained within the article.
